# Sexual and reproductive health of in-transit migrant women en route to the United States: a mixed-methods study in Ciudad Juárez, Mexico

**DOI:** 10.1186/s44263-025-00180-8

**Published:** 2025-07-07

**Authors:** Silvana Larrea-Schiavon, César Infante, Jay Graham, Sylvia Guendelman

**Affiliations:** 1 Interdisciplinary Department, School of Public Health, University of California Berkeley, Berkeley, USA; 2 Health Systems Research Center, National Institute of Public Health, Cuernavaca, Mexico; 3 Division of Environmental Sciences, School of Public Health, University of California Berkeley, Berkeley, USA; 4 Wallace Center for Maternal, Child and Adolescent Health, School of Public Health, University of California Berkeley, Berkeley, USA

**Keywords:** Sexual and reproductive health, Migrant in transit, Undocumented in transit migrant women, Utilization of Healthcare services, Mexico, Central American migrants

## Abstract

**Background:**

The number of undocumented in-transit migrant women (UITMW) traveling through Mexico to the U.S. is increasing, with longer stays in Mexico. We explore how UITMW’s sexual and reproductive health (SRH) needs and service utilization for these needs vary by time spent in Mexico and availability of instrumental social support. We also identify barriers to care and propose actionable steps to improve service delivery.

**Methods:**

We conducted a sequential quantitative–qualitative mixed-methods study in Ciudad Juárez, Mexico. It includes a secondary analysis of a 2021 survey of 252 UITMW and a primary analysis of 31 stakeholder interviews in 2023, that elaborated on the survey findings. Guided by Andersen’s Behavioral Model of Health Care Utilization, we performed bivariate analyses to assess SRH needs and service utilization by time spent in Mexico and instrumental social support. Multivariate logistic regression models estimated unadjusted (ORs), adjusted odds ratios (aORs), and 95% confidence intervals (95% CIs) to assess these associations and the modifying effect of instrumental social support. For the qualitative component, we applied a framework analysis structured around four key themes from the quantitative findings to contextualize results and identify barriers and actionable solutions.

**Results:**

Nine in ten UITMW experienced at least one SRH need, yet only 33.6% accessed SRH services. Interviewees cited fear of organized crime, government authorities, and constant mobility as key barriers. While longer stays in Mexico were initially associated with higher SRH utilization, this was no longer significant when adjusting for covariates (aOR 1.87; 95% CI 0.83–4.19). However, among women without instrumental social support, longer stays significantly increased the odds of SRH service utilization (aOR 6.99; 95% CI 1.42–34.45). This pattern may reflect greater challenges accessing care earlier in their stay. Qualitative findings suggest that instrumental social support may facilitate service utilization by enabling childcare, sharing information, and fostering connections.

**Conclusions:**

UITMW face SRH needs and multiple barriers to care while in Mexico. Utilization is particularly challenging for UITMW who experience sexual violence and lack instrumental social support. Understanding the factors influencing the health needs and SRH utilization of UITMW can help Mexico’s health system plan effective interventions.

**Supplementary Information:**

The online version contains supplementary material available at 10.1186/s44263-025-00180-8.

## Background

The influx of undocumented migrants traveling through Mexico en route to the United States (U.S.) has been surging, and their stays in Mexico have been growing longer [1]. Migrants temporarily staying in a country that is not their final destination are considered “in-transit migrants” [2]. Between 2011 and 2022, Mexico experienced a seven-fold increase in the number of undocumented in-transit migrants traveling to the U.S. as poverty, crime, violence, and natural disasters increased in countries of origin [3]. In 2023, the Mexican immigration agency reported a total of 782,176 detentions of undocumented migrants, of which 31% were women and 31.6% came from Central America [3].

Immigration policies implemented by the U.S. and the Mexican governments to reduce the number of undocumented in-transit migrants in Mexico have made travel to the Mexican northern border more challenging [4, 5]. Furthermore, the presence of organized crime groups in the migration routes has made this a very perilous journey, leading to multiple abuses and disappearances [6].

Many who reach the northern border remain stuck in Mexican cities, as increasingly restrictive U.S. policies limit entry [4, 5]. The proportion of migrants staying in Mexico for over a month rose from 3.8% in 2009 to 31.6% in 2019 [7]. A 2020 report by Doctors Without Borders characterized the situation as a humanitarian crisis marked by precarious living conditions, lack of support, and frequent rights violations, such as ill-treatment by authorities and arbitrary detentions [8, 9].

For undocumented in-transit migrant women (UITMW), the journey is even more dangerous. Compared to men, they face heightened exposure to sexual violence and may resort to survival sex for food, shelter, and protection [10–12]. These conditions may affect their sexual and reproductive health (SRH). The latter refers to a whole-person approach that encompasses the need for safe and respectful sexual relationships and protection from sexual abuse; access to effective and safe contraception; the need to hygienically and comfortably manage menstrual periods; protection against sexually transmitted infections; prevention of unintended pregnancy; and effective pregnancy care [13, 14].

Evidence on the SRH needs of UITMW in Mexico from peer-reviewed public health journals [15–18], research institutions [19], and international agencies [20] shows that UITMW experience numerous needs, including sexually transmitted infections, unwanted or unplanned pregnancies, inadequate pre-natal and obstetric care, and unsafe abortions. Studies by Vázquez-Quesada et al. and Letona et al. specifically highlight the unmet menstrual health needs experienced by UITMW during transit, including severe menstrual pain and a lack of menstrual hygiene supplies [18, 19]. These studies employ quantitative analyses using large samples and longitudinal data [15–17, 19], qualitative research based on in-depth interviews providing rich contextual insights [18], and real-time monitoring of migration trends [20], though some studies may face limitations in representativeness [18–20] and desirability biases [18, 19].

Mexico has formally recognized healthcare as a right for all, regardless of immigration status, through legal reforms and health programs that expand access to care [21–23]. Nonetheless, studies conducted in Mexico after the implementation of these policies indicate that challenges in providing health services to undocumented in-transit migrants persist across three levels [10, 19, 24–30]. At the individual level, UITMW often lack knowledge of their rights and face financial and mobility constraints [31–33]. At the institutional level, service providers report limited resources and training to offer appropriate care [34]. At the structural level, health and migration policies are geared toward short-term stays, failing to address the needs arising from prolonged transit situations [35].

Social support plays a crucial role in mitigating these barriers, serving as a key form of “social capital” [36, 37]. Research indicates that informal support networks facilitate greater utilization of SRH services by offering emotional encouragement, sharing vital information, and assisting with health system navigation [38–40].

Despite this growing body of literature, gaps remain. It is unclear how the duration of stay in Mexico impacts UITMW’s SRH needs and service utilization. Short stays may hinder utilization due to time constraints, whereas longer stays could make access to care increasingly necessary, as unmet SRH needs accumulate and become more urgent. In addition, while studies report on the benefit of generalized social support, the specific role of instrumental support (e.g., financial assistance and material resources) has not been sufficiently explored in contexts of high in-transit migration, such as Mexico.

To address these gaps, we examined the SRH needs and health service utilization reported by UITMW, exploring how SRH needs and utilization vary by time spent in Mexico and by the availability of instrumental social support. Based on evidence from destination countries, we hypothesized that (1) SRH needs are prevalent among UITMW and increase with time spent in Mexico, leading to greater SRH service utilization; (2) despite their SRH needs, SRH service utilization is low among UITMW and they face barriers to care; and (3) the availability of instrumental social support facilitates earlier utilization of SRH services. Subsequent in-depth qualitative interviews with multiple stakeholders who work with migrant populations explored the mechanisms through which these factors influence SRH needs and service utilization, shedding light on current barriers to care and actionable steps to improve service delivery.

## Methods

### Study design

We conducted a sequential mixed-methods study in Ciudad Juárez, Mexico, consisting of a secondary data analysis of a quantitative survey conducted in 2021, which captured the direct experiences of UITMW staying in shelters, followed by qualitative interviews with service providers, decision-makers, and migration experts in 2023.

Both study components examined UITMW’s SRH needs and service utilization, but from different perspectives and at different points in time. We first analyzed the quantitative findings, then leveraged the qualitative interviews to elaborate on, complement, and clarify the survey results [41]. With the purpose of complementarity, we used the interviews to provide contextual depth, placing the quantitative findings within the broader realities of UITMW’s SRH needs and healthcare access while in transit through Mexico [41]. The interviews also helped identify barriers to SRH care and recommendations to improve care.

**Fig. 1:**
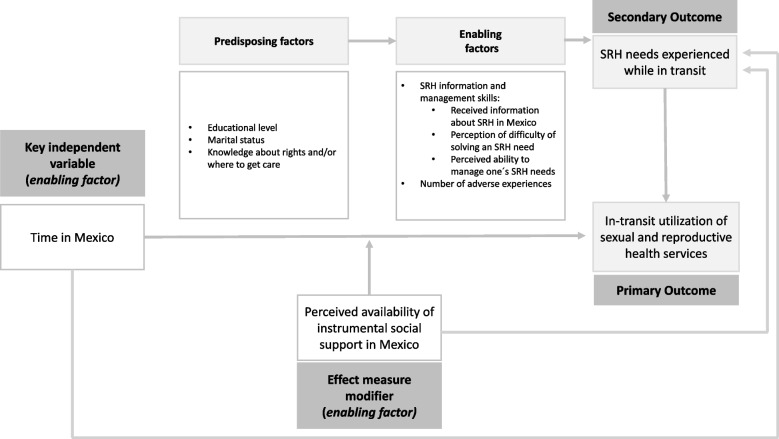
Factors associated with utilization of sexual and reproductive health services by undocumented in-transit migrant women. Conceptual model

Our work is guided by Andersen’s Behavioral Model of Health Care Utilization which proposes that healthcare utilization is based on three factors: the individual’s predisposition to use health services which includes demographic, social, and beliefs influencing care seeking behaviors; enabling resources or hindering factors that impede use; and the actual need-for-care, considered a proximal determinant of utilization [42]. As shown in Fig. 1, our primary outcome is SRH service utilization among UITMW. We also examine SRH needs while in transit as a secondary outcome. The key independent variable is duration of stay in Mexico, and we explore the potential effect modification of this relationship by perceived availability of instrumental social support. Additionally, we consider predisposing and enabling factors that may shape these associations. The predisposing factors included were educational level, marital status, and knowledge about rights and SRH service locations in Mexico. The enabling factors assessed whether a woman had received SRH information in Mexico, whether she perceived difficulty in accessing SRH services, her perceived ability to manage SRH needs, and situations increasing the likelihood of UITMW experiencing SRH needs (e.g., adverse events).

### Quantitative component

De-identified data were obtained from the “Sexual and Reproductive Health Needs and Care of Migrant Women in Mexico” survey. The lead author (SL) formally requested access to the dataset from the Population Council. A data sharing agreement was subsequently signed between both parties to govern the terms of data access and use. Employing a convenience sampling strategy, the survey was conducted between June and September of 2021 by the Population Council and the Colegio de la Frontera Norte in six migrant shelters in Ciudad Juárez [19]. Ciudad Juárez, a city in the state of Chihuahua that shares borders with El Paso, Texas, is one of the main crossing points to the U.S. [7]. In 2022, two out of every ten undocumented migrants who were detained in the U.S. had crossed the border through the state of Chihuahua [43].

A total of 266 in-transit undocumented adult migrant women staying in shelters answered the survey [19]. Participants who met three selection criteria were included in our analysis: (1) their country of origin was in Central America; (2) they identified as cis-gender women; and (3) they were between 18 and 49 years old, yielding an analytic sample of 252 participants. Verbal consent was obtained from all survey participants. Further details about the survey, dataset, and codebook are available in the Harvard Dataverse repository [44].

#### Measures

Self-reported utilization of SRH services, our *primary outcome*, was categorized as a binary variable (used vs. did not use any SRH service provided in a public or private clinic/hospital, in a pharmacy-based clinic, a non-governmental organization (NGO) or a migrant shelter since the respondent’s arrival in Mexico). We defined SRH services as those that address UITMW’s sexual and reproductive health concerns, as measured by the question “Did you receive care for [specific SRH need]?”, including medical care for symptoms related to reproductive tract infections, menstrual pain, and medical care received at any point during pregnancy.

Perceived SRH needs, our *secondary outcome*, is based on a recognized global definition of SRH needs [13, 14]. This outcome was identified from survey questions that queried about the presence of menstrual health symptoms and management (e.g., severe menstrual pain, irregular periods, and lack of access to menstrual products), reproductive tract infections-related symptoms (e.g., itching, ulcers, abnormal vaginal discharge), sexual violence (defined as inappropriate touching), contraceptive use, and pregnancy. Using this information, we created two variables: (1) reported at least one SRH need while in transit versus none and (2) the total number of SRH needs experienced while in transit. The survey questions used to measure these variables are provided in Additional File 1.

Time spent in Mexico, an *enabling factor of health service utilization*, was our key independent variable. Consistent with Mexican national surveys on migration flows [7], we categorized time spent in Mexico as ≤ 15 days (short stay), between 16 and 30 days (intermediate stay), and > 30 days (longer stay). We categorized time spent in Mexico as a three-level variable to account for the variable’s non-normal distribution and the low participant numbers in several categories.

Perceived availability of instrumental social support, another enabling factor, was used to test whether it modified the relationship between time spent in Mexico and SRH service utilization. It was defined as having at least one person in Mexico who could lend the respondent money in case of financial need and/or someone who could support with childcare if needed [36]. We coded this variable as 0 if participants answered “No” to both questions and 1 if they answered “Yes” to at least one.

Potential covariates consisted of *predisposing factors and other enabling factors.* Educational level was coded as basic education versus high school or higher. Marital status was categorized as single, married but not currently living with partner, and married and currently living with partner. Knowledge about health rights and access to SRH services in Mexico was assessed using two survey questions: (1) “According to what you know, do migrants who travel without papers/documents have the right to receive health care in Mexico?” and (2) “If you had a health problem today, do you know of any place where you could get care that you consider trustworthy?”. The variable was coded as 0 if participants answered “No” to both and 1 if they answered “Yes” to at least one.

*Other enabling* factors include a cluster of variables related to SRH information and management skills. We defined SRH management skills as perceived abilities that can hinder or empower individuals to seek appropriate care, including knowledge acquisition, perceived barriers to care, and coping strategies such as seeking medical care and utilizing self-care interventions when appropriate [45, 46]. We kept this cluster as three uncorrelated variables, including receiving information about SRH and SRH services in Mexico (e.g., did they receive information about birth control methods, how to prevent sexually transmitted infections, and gender-based violence in Mexico), perception of the difficulty of solving an SRH need, and perceived ability to manage one’s SRH needs. We built the continuous variable “perception of the difficulty of solving an SRH need” by summing the number of SRH needs women perceived as difficult to address while in Mexico. The survey assessed difficulty across nine SRH needs, including menstrual health, pregnancy prevention, prenatal care, delivery care, postpartum care, preventing sexually transmitted infections, treating sexually transmitted infections, sexual violence, and abortion. Perceived ability to manage one’s SRH needs is a binary variable (Yes/No) based on the survey question: “Do you currently feel that you are able to take care of your women’s health (SRH) needs as you would like?” Finally, the number of adverse events experienced while in transit was measured as a categorical variable (less than 5 risks, between 5 and 8 risks, and more than 8 risks). The survey assessed experiences such as economic insecurity, lack of access to food or water, unsafe shelter or housing, humiliation through degrading actions, threats and intimidation inducing fear of harm, physical violence, and crime-related incidents such as robbery, kidnapping, and coerced substance use.

#### Data analysis

We first conducted descriptive statistics of selected socio-demographic characteristics of the survey participants, as well as their perceived SRH needs and the SRH utilization patterns, stratified by time spent in Mexico and the availability of instrumental social support. To test our hypothesis that SRH service utilization is low among UITMW, we explored how many UITMW with a SRH need utilized SRH services. We also conducted bivariate analyses of the association between the predisposing and enabling factors, and (1) time spent in Mexico, (2) perceived availability of instrumental social support, and (3) utilization of SRH services. A *p* value ≤ 0.05 was considered statistically significant, although we also highlighted associations with a *p* value ≤ 0.10 due to small sample sizes (< 100 responses). We report results as medians and ranges for continuous variables and frequencies and percentages for categorical variables. We performed additional Chi-squared tests on each pair of groups for those variables that were statistically significant (e.g., married women who stayed in Mexico > 30 days vs. married women who stayed in Mexico ≤ 15 days).

We used multivariate logistic regression models to test our hypothesis that women staying longer in Mexico use more services than those staying shorter. We calculated unadjusted odds ratios (ORs) and adjusted odds ratios (aORs), as well as 95% Confidence Intervals (CIs). Before entering variables into the models, we tested for collinearity to ensure that the predisposing and enabling variables were not significantly related to each other at *p* value ≤ 0.05. “Migrating with children” was excluded from the model since only 13 women reported traveling without children.

Finally, we tested whether the perceived availability of instrumental social support modified the association between SRH service utilization and time spent in Mexico. We examined the effect measure modification in both multiplicative and additive scales, following Knol and VanderWeele’s recommendations for presenting analyses of effect measure modification and interaction [47]. The narrative presents the results in the multiplicative scale. Tables with the absolute difference in the probability of utilizing SRH services (additive scale) are included in Additional File 2.

We conducted all analyses in Stata 18.0 (StataCorp LLC, College Station, TX, USA) [48].

### Qualitative component

This study follows the Consolidated Criteria for Reporting Qualitative Studies (COREQ) checklist [49], which is included in Additional File 3. We used the qualitative results to elaborate, clarify, and contextualize our quantitative findings from the survey with UITMW.

We used purposive sampling to recruit key informants with expertise in SRH and migration [50]. Through word-of-mouth, we compiled a list of potential participants working in Ciudad Juárez, Chihuahua City, or at the federal level, providing services or shaping policies related to SRH and migration. Recruitment began in April 2023. The main author (SL)—a cisgender woman and Doctor of Public Health student at the time, with over seven years of experience in migration and health research in Mexico—contacted potential participants via email, answered any questions, and explained the study’s goals and reasons for conducting the research prior to scheduling interviews.

Between August and November 2023, SL conducted 31 interviews with 36 key informants. Participants represented government (federal, state, and municipal), public-sector health services, shelters, non-governmental organizations, and international agencies. We stopped recruiting participants once we reached informational redundancy. Only one of the recruited participants, a federal decision-maker, declined to participate.

Based on our conceptual model, we developed three interview guides, one for each group of participants, which are included in Additional File 4. The guides explored themes regarding UITMW’s SRH needs (e.g., what SRH needs are more prevalent and why) and the barriers and facilitators to providing SRH services for this population. The guides underwent iterative development throughout the data collection process to reflect new information learned from participants and the quantitative results.

SL conducted one-on-one interviews via Zoom, over the phone, or in person at the participant’s workplace, based on individual preferences. Each interview lasted approximately 90 min and was audio-recorded. No repeat interviews were conducted. Following each interview, SL wrote a post-interview memo. Participants received compensation of $10.00 (~ MXN 200.00) or a keychain of equivalent value. SL listened to each interview and cleaned and de-identified the transcripts. We then uploaded the transcripts to the MAXQDA qualitative analysis software (VERBI Software, Berlin, Germany) [51].

#### Data analysis

Following Klingberg et al.’s framework analysis approach [52], SL and CI applied a deductive-inductive coding process. A priori codes were developed based on our conceptual framework and quantitative findings. Data were organized in an Excel matrix with participants as rows and a priori codes as columns. Additional inductive codes emerged during coding, and relevant information was summarized within the matrix. We then structured qualitative findings around key themes identified from the quantitative analysis, selecting representative quotes to illustrate results. SL and CI coded the first two interviews and refined the initial codebook, with SL coding the remaining transcripts. SL, CI, and SG held regular discussions to refine emerging themes. The final codebook is included in Additional File 5. We shared a preliminary version of the results with participants during a workshop held in Ciudad Juárez in May 2024. Participants endorsed the findings, affirming that the results accurately reflected their experiences working with these populations. No changes were made as a result of this meeting.

### Integration of the qualitative and quantitative components

To integrate the findings from both components, we first analyzed the survey data and identified four key quantitative findings related to SRH needs and service utilization among UITMW. These findings informed the thematic organization of the qualitative analysis. Specifically, interview transcripts were reviewed to further explore and contextualize each of the four findings. This approach allowed for a complementary interpretation of results, using qualitative data to enrich the understanding of patterns identified in the quantitative analysis. Accordingly, the results section is structured by first presenting the survey findings, followed by the qualitative findings, which are organized around and integrated with the four key themes emerging from the quantitative analysis.

## Results

### Survey results

#### Characteristics of survey participants

The median age of participants was 28 years (range 18–49 years). Eight out of ten UITMW were in Mexico with their children. Just over half of the study population was from Honduras and 64.7% reported fleeing violence as the main reason for leaving their country of origin. Of the 252 UITMW, 41.3% had been in Mexico for ≤ 15 days, 30.9% between 16 and 30 days, and 27.8% for more than 30 days (range 30 days to 5 years) (Table 1).

**Table 1:** Characteristics of undocumented in-transit migrant women in Mexico

Characteristics	Undocumented in-transit migrant women (*N* = 252)	
	***n* or median**	**% or range**
Age (median and range)	28	18–49
Migrating with their children (n and %)	215	85.3
Country of Origin (*n* and %)		
Honduras	138	54.8
Guatemala	83	32.9
El Salvador	22	8.7
Nicaragua	9	3.6
Main reason for leaving their countries of origin (n and %)		
Fleeing violence	163	64.7
Fleeing gender-based violence	39	15.5
Fleeing political persecution	10	4.0
Better economic/employment opportunities	70	27.8
Other	9	3.6
Time spent in Mexico (*n* and %)		
≤ 15 days	104	41.3
Between 16 and 30 days	78	30.9
More than 30 days	70	27.8

#### SRH needs and SRH service utilization

Even though 91.0% (95% CI 86.6, 94.1) of UITMW experienced at least one SRH need since arriving in Mexico, most did not seek healthcare for their SRH needs (66.4%; 95% CI 60.0, 72.5) (Table 2). Among women who experienced sexual violence during transit (19.4%; 95% CI 14.7, 24.9), only 8% (95% CI 2.3, 19.6) utilized health services (results not shown).

**Table 2:** Sexual and reproductive health (SRH) needs and service utilization, by time spent in Mexico and perceived availability of instrumental social support

SRH needs and SRH service utilization	Total *N* = 252 (100%)	Time spent in Mexico				Perceived availability of instrumental social support		
		** ≤ 15 days** ***n* = 104 (41.3%)**	**16–30 days** ***n* = 78 (30.9%)**	** > 30 days** ***n* = 70 (27.8%)**	***p* value**	**Yes** ***n* = 150 (64.1%)**	**No** ***n* = 84** **(35.9%)**	***p* value**
	***n*, %** **[95% CI]**	***n*, %** **[95% CI]**	***n*, %** **[95% CI]**	***n*, %** **[95% CI]**		***n*, %** **[95% CI]**	***n*, %** **[95% CI]**	
Menstrual health and management needs	222, 88.1% [83.4, 91.8]	89, 85.6% [77.3, 91.7]	67, 85.9% [76.2, 92.7]	66, 94.3% [86.1, 98.4]	0.10	129, 86.0% [79.4, 91.1]	75, 89.3% [80.6, 95.0]	0.47
Reproductive tract infections-related symptoms	136, 54.0% [47.6, 60.2]	50, 48.1% [38.2, 58.1]	43, 55.1% [43.4, 66.4]	43, 61.4% [49.0, 72.8]	**0.08**	84, 56.0% [47.7, 64.1]	43, 51.2% [40.0, 62.3]	0.48
Sexual violence	49, 19.4% [14.7, 24.9]	16, 15.4% [9.1, 23.8]	16, 20.5% [12.2, 31.2]	17, 24.3% [14.8, 36.0]	0.15	31, 20.7% [14.5, 28.0]	15, 17.8% [10.3, 27.7]	0.60
Contraception	10, 4.0% [1.9, 7.2]	3, 2.9% [0.6, 8.2]	0	7, 10.0% [4.1, 19.5]	**0.05**	4, 2.7% [0.7, 6.7]	6, 7.1% [2.7, 14.9]	0.12
Pregnancy	15, 5.9% [3.4, 9.6]	7, 6.7% [2.7, 13.4]	0	8, 11.4% [5.1, 21.3]	0.30	6, 4.0% [1.5, 8.5]	9, 10.7% [5.0, 19.4]	**0.04**
Perceived at least one SRH need while in transit	229, 91.0% [86.6, 94.1]	95, 91.3% [84.2, 96.0]	68, 87.2% [77.7, 93.7]	66, 94.3% [86.0, 98.4]	0.61	134, 89.3% [83.3, 93.8]	78, 92.9% [85.1, 97.3]	0.38
Number of SRH needs perceived while in transit median (range)	4 (0–12)	4 (0–12)	3 (0–12)	4.5 (0–12)	**0.02**	4 (0–12)	4 (0–12)	0.63
Utilized SRH services, among those with SRH needs	77, 33.6% [27.5, 40.1]	31, 32.6% [23.4, 43.0]	15, 22.1% [12.9, 33.8]*	31, 47.0% [34.6, 59.7]*	**0.08**	42, 31.3% [23.6, 39.9]	28, 35.9% [25.3, 47.6]	0.22

Bi-variate analyses were conducted to test for differences by time spent in Mexico; (*) Chi-squared tests between groups with p-values ≤ 0.05

The most reported SRH needs were menstrual health and management (88.1%; 95% CI 83.4, 91.8) and reproductive tract infections-related symptoms (54.0%; 95% CI 47.6, 60.2) (Table 2). Women with longer stays in Mexico were more likely to report SRH needs (*p* = 0.02) and tended to use SRH services more frequently than those with intermediate and shorter stays (≤ 15 days) (*p* = 0.08) (Table 2). However, the availability of instrumental social support was not associated with either the number of SRH needs (*p* = 0.63) or the use of health services (*p* = 0.22).

#### Predisposing and enabling factors

Most UITMW had only primary education (66.3%), were single (50.4%), and knew their SRH rights and where to access care in Mexico (65.9%). Women perceiving instrumental social support had higher education levels (*p* = 0.01) and greater awareness of their rights and available SRH services (*p* = 0.006) (Table 3). While 56.3% of UITMW felt able to manage their SRH needs, most perceived that solving their needs in Mexico is difficult (Table 3). Women with longer stays in Mexico and those without instrumental social support reported experiencing 5–8 adverse events more frequently than those with shorter stays (*p* < 0.001) or those who perceived having instrumental social support (*p* = 0.03). SRH service utilization was significantly associated with the perception of difficulty (*p* = 0.005) and the ability to manage one’s SRH needs (*p* = 0.02).

**Table 3:** Predisposing and enabling factors, by time spent in Mexico, perceived availability of instrumental social support, and utilization of sexual and reproductive health (SRH) services

Predisposing and enabling factors	Total *N* = 252 (100%)	Time spent in Mexico				Perceived availability of instrumental social support			Utilized a SRH service		
		** ≤ 15 days** ***n* = 104 (41.3%)**	**16–30 days** ***n* = 78 (30.9%)**	** > 30 days** ***n* = 70 (27.8%)**	***p* value**	**Yes** ***n* = 150 (64.1%)**	**No** ***n* = 84** **(35.9%)**	***p* value**	**Yes** ***n* = 79 (31.4%)**	**No** ***n* = 173** **(68.6%)**	***p* value**
		***n* (%)**	***n* (%)**	***n* (%)**		***n* (%)**	***n* (%)**		***n* (%)**	***n* (%)**	
**Predisposing factors**											
Educational level											
Basic education	167 (66.3)	67 (64.4)	52 (66.7)	48 (68.6)	0.63	93 (62.0)	66 (78.6)	**0.01**	54 (68.3)	113 (65.3)	0.54
High school or higher	84 (33.3)	36 (34.6)	26 (33.3)	22 (31.4)		57 (38.0)	17 (20.2)		24 (30.5)	60 (34.7)	
Missing	1 (0.40)	1 (1.0)	0	0		0	1 (1.2)		1 (1.2)	0	
Marital status											
Single	127 (50.4)	63 (60.6)*	35 (44.9)	29 (41.4)	**0.005**	78 (52.1)	40 (47.6)	0.46	33 (41.8)	94 (54.3)	**0.06**
Cohabitating or married and currently living with partner	79 (31.4)	26 (25.0)	24 (30.8)*	29 (41.4)*		44 (29.3)	29 (34.5)		30 (38.0)	49 (28.4)	
Cohabitating or married but not currently living with partner	42 (16.6)	12 (11.5)	18 (23.1)	12 (17.2)		26 (17.3)	15 (17.9)		15 (19.0)	27 (15.6)	
Missing	4 (1.6)	3 (2.9)	1 (1.2)	0		2 (1.3)	0		1 (1.2)	3 (1.7)	
Knows about rights and/or where to get care	166 (65.9)	64 (61.5)	49 (62.8)	53 (75.7)	**0.07**	108 (72.0)	46 (54.8)	**0.006**	54 (68.4)	112 (64.7)	0.79
Missing	24 (9.5)	8 (7.7)	13 (16.7)	3 (4.3)		12 (8.0)	8 (9.5)		6 (7.6)	18 (10.5)	
**Enabling factors**											
Received information about SRH and/or SRH services in Mexico	26 (10.3)	5 (4.8)	7 (9.0)	14 (20.0)*	**0.002**	14 (9.3)	9 (10.7)	0.73	12 (15.2)	14 (8.1)	0.12
Missing	8 (3.2)	2 (1.9)	3 (3.8)	3 (4.3)		3 (2.0)	2 (2.4)		0	8 (4.6)	
Perceived they have instrumental social support while in Mexico	150 (59.5)	63 (60.6)	50 (64.1)	37 (52.8)	0.26	NA	NA	NA	42 (53.2)	108 (62.4)	0.22
Missing	18 (7.2)	10 (9.6)	2 (2.6)	6 (8.6)		NA	NA		7 (8.8)	11 (6.4)	
Perception of difficulty of solving SRH needs (median [range])	8 (0–9)	8 (0–9)	8 (0–9)	7 (1–9)	0.88	8 (0–9)	8 (1–9)	0.60	7 (0–9)	8 (1–9)	**0.005**
Perceived they have the ability to manage their SRH needs	142 (56.3)	58 (55.8)	46 (59.0)	38 (54.3)	0.73	88 (58.7)	43 (51.2)	0.31	55 (69.6)	87 (50.3)	**0.02**
Missing	22 (8.7)	11 (10.6)	5 (6.4)	6 (8.6)		10 (6.7)	7 (8.3)		3 (3.8)	19 (11.0)	
Number of adverse experiences during transit											
< 5 risks	92 (36.5)	50 (48.1)*	29 (37.2)	13 (18.5)	**0.000**	62 (41.3)	23 (27.4)*	**0.03**	23 (29.1)	69 (39.9)	0.10
5–8 risks	123 (48.8)	41 (39.4)	36 (46.1)	46 (65.7)*		68 (45.3)	49 (58.3)*		45 (57.0)	78 (45.1)	
9–12 risks	37 (14.7)	13 (12.5)	13 (16.7)	11 (15.7)		20 (13.4)	12 (14.3)		11 (13.9)	26 (15.0)	

Bi-variate analyses were conducted to test for differences by time spent in Mexico, perceived availability of social support, and utilization of SRH services; (*) Chi-squared tests between groups with *p* values ≤ 0.05

#### Time spent in Mexico and utilization of SRH services among women with and without instrumental social support

There was a positive, although marginally, significant association between time spent in Mexico and utilization of health services. Women who had been in Mexico for more than 30 days had almost twice the odds (OR 1.87; 95% CI 0.99–3.52) of using SRH services when compared to women who had been in the country for ≤ 15 days (Table 4, Model1).

**Table 4:** Crude and adjusted odds ratios and 95% CI of time spent in Mexico and sexual and reproductive health (SRH) service utilization, further stratified by perceived availability of instrumental social support

Variables	Total sample		Perceived availability of instrumental social support	
	**Unadjusted**	**Adjusted**	**Yes**	**No**
	**Model 1** OR (95% CI)	**Model 2** aOR (95% CI)	**Model 3** aOR (95% CI)	**Model 4** aOR (95% CI)
	***n* = 252**	***n* = 195**	***n* = 127**	***n* = 68**
Time in Mexico				
≤ 15 days	REF	REF	REF	REF
16–30 days	0.66 (0.33–1.30)	0.72 (0.32–1.65)	0.81 (0.30–2.16)	0.36 (0.05–2.80)
> 30 days	**1.87 (0.99**–**3.52)^**	1.87 (0.83–4.19)	1.00 (0.33–3.04)	**6.99 (1.42**–34.45)*
**Predisposing factors**				
Educational level				
Basic education	–	REF	REF	REF
> Basic education	–	0.84 (0.39–1.80)	1.00 (0.38–2.66)	0.96 (0.19–4.80)
Marital status				
Single and not currently living with a partner	–	REF	REF	REF
Cohabitating or married but not currently living with partner	–	1.51 (0.59–3.85)	1.19 (0.36–3.97)	**5.43 (0.79**–**37.0)^**
Cohabitating or married and currently living with partner	–	**2.08 (0.98**–**4.44)^**	1.54 (0.57–4.17)	3.35 (0.75–14.94)
Knowledge about rights and/or where to get care				
Does not know about rights	–	REF	REF	REF
Knows about rights	–	0.97 (0.45–2.11)	1.30 (0.43–3.95)	0.54 (0.13–2.16)
**Enabling factors**				
Received information about SRH and/or SRH services in Mexico				
No	–	REF	REF	REF
Yes	–	1.36 (0.45–4.12)	1.29 (0.26–6.42)	1.52 (0.23–10.06)
Perceived availability of instrumental social support				
No	–	REF	–	–
Yes	–	0.91 (0.45–1.86)	–	–
Perception of difficulty of solving an SRH need	–	**0.78 (0.67**–0.91)*	**0.71 (0.58**–0.87)*	0.87 (0.62–1.21)
Perceived ability to manage one´s SRH needs				
No	–	REF	REF	REF
Yes	–	**2.19 (1.05**–4.60)*	**2.63 (0.99**–**7.03)^**	2.72 (0.65–11.35)
Number of adverse events experienced during transit				
< 5 risks	–	REF	REF	REF
5–8 risks	–	0.99 (0.45–2.14)	1.23 (0.45–3.41)	0.91 (0.21–3.93)
9–12 risks	–	0.90 (0.28–2.84)	1.10 (0.25–4.88)	1.68 (0.16–17.82)
Number of perceived SRH needs while in transit	–	1.05 (0.94–1.17)	**1.13 (0.98**–**1.31)^**	0.83 (0.66–1.06)
***Model Pseudo R2***	–	0.107	0.131	0.214

^*^p value ≤ 0.05; ^*p* value ≤ 0.10

When controlling for several predisposing, enabling factors, and perceived SRH needs, the association between > 30 days vs. ≤ 15 days spent in Mexico and utilization of health services was not statistically significant (aOR 1.87; 95% CI 0.83, 4.19) (Table 4, Model2). In this model, SRH management skills were significantly associated with SRH service utilization. Specifically, women who perceived greater difficulty in addressing their SRH needs (vs. less difficulty) had lower odds of utilizing health services (aOR 0.78; 95% CI 0.67,0.91). In contrast, women who perceived themselves as able to manage their SRH needs had higher odds of utilizing health services compared to those who did not perceive having this ability (aOR 2.19; 95% CI 1.05–4.60).

When stratifying by perceived instrumental social support, we found that among women without instrumental social support, those who stayed in Mexico for > 30 days had 6.99 times higher odds of using health services compared to those with stays of ≤ 15 days (CI 95% 1.42, 34.45), after adjusting for all the other covariates (Table 4, Model 4). This association may reflect that, although all UITMW are exposed to increasingly adverse living conditions and SRH needs the longer they remain in Mexico, those without instrumental social support face additional barriers to care—barriers that may be harder to overcome without access to childcare, information, or social networks (Table 4, Model 3).

In summary, there are four main findings from the survey: (1) SRH needs are prevalent among UITMW and increase with longer stays in Mexico; (2) SRH service utilization is low among UITMW; (3) SRH management skills are associated with SRH service utilization; and (4) Instrumental social support modifies the association between time in Mexico and SRH service utilization.

### Qualitative findings

We present the main findings grouped according to the four key results of our quantitative analysis. Quotes that back up the qualitative findings are reported in Table 5.

**Table 5:** Key findings from the interviews and supportive quotes

Key survey findings from UITMW	Key findings from service providers, decision-makers, and subject matter experts	Quotes
SRH needs are prevalent among UITMW and increase with longer stays in Mexico	• In-transit migrant women are spending at least a month in Mexico while awaiting their opportunity to cross to the US • Women´s SRH needs are dependent on the context of their journey • Women´s perception of SRH needs increases as they spend more time in a place, especially if they feel safe or settled • New SRH needs might arise while they are waiting for an opportunity to cross to the US, associated to exposure to risks, changes in migration plans (e.g., starting a family), or because of lack of access to health services	“*As the type of people who come and the route they take change, so are the risks they are exposed to along this journey. Women who migrate accompanied, women who migrate by themselves. Some of them make friends along the way and everything, and they support each other. But the people who come alone or who take the risk by themselves, well, that is where many risks and complications of this type arise*”. **Social worker, public-sector hospital, 40 yo, female, Ciudad Juárez** “*Let’s say that they already had some health condition in their country of origin, but they never sought care. So, they start, they leave their country of origin, they are in transit and their child gets sick, and they [the women] feel sick, but the first thing to take care of is their son, or I don’t know, their daughter or something like that, right? So, when they arrive and, let’s say, they have that possibility of settling, all that stuff [health needs] start to appear, right? In other words, I talk to them, and they say, “Oh no, because suddenly I have this and this”. So, I say, “Okay, then let’s take you to get checked”. But from there, it is like if a Pandora’s box opens and a lot of things appear that she surely knew she had, but that she never followed up on*”. **Social worker, NGO, 33 yo, female, Mexico City** “*It´s good that many of the migrants are sheltered. But if you saw how the streets are full of migrants, of women with their small children, some of them very little, still breastfeeding, some of them being hold by the hands, who are begging for money, who are cleaning the windows, who… These women become a loot, right? They are harassed by men driving their cars… In other words, in many ways, some, not all, but some are forced into sex work for money. And some others, men and women, are being used by the mafias to store drugs, to sell, eh, in short, for many things, right?*”. **Local decision-maker, government institution, 52 yo, female, Ciudad Juárez**
SRH service utilization is low among UITMW	• Women are afraid of denouncing instances of sexual violence, and they do not trust law enforcement institutions • Being on the move makes it difficult to utilize care and denounce instances of sexual violence • Lack of timely access to SRH services has an impact on SRH outcomes • Staying in migrant shelters is a key facilitator of health care utilization, although some faith-based shelters tend to regulate SRH behaviors and access to certain SRH services • Shelters and NGOs accompany women to their medical appointments to reduce the likelihood of denial of services • Creation of an inter-agency committee to address the health needs of migrant populations in Ciudad Juárez	*“The thing I have heard the most from women is that they know they* [organized crime] *have eyes and ears everywhere, so they are going to know* [that they denounce the sexual violence]*, right? Even if they are going to, well, if they go to the prosecutor’s office, they* [organized crime] *will know…”.* **Lawyer, international NGO, 32 yo, female, Ciudad Juárez** “*I diagnose pregnant women in the emergency room after the third trimester. They don’t have an ultrasound. In fact, they have never had tests done until I do them […]. There was no folic acid. Well, we don´t know what diseases they have, nor do they know what diseases they may have. We have diagnosed many with syphilis, with trichomonas, with HIV […]. The Neonatal Intensive Care Unit is saturated because, since there was no prenatal control, things get complicated for us. They are born prematurely, and we already have a complicated mother and a complicated premature baby*”. **OBGyN, public-sector hospital, 35 yo, female, Ciudad Juárez** “*Now, I am going to tell you another big difference, in the shelters they* [women] *will have help from many* [people], *of different kind, from organizations, foundations, people, because there are local people who go to the shelters and bring things. And to those who are on the street, who? Well, no*”. **Service provider, NGO, 34 yo, male, Ciudad Juárez** “*Also, if they do not request it* [SRH service]*, then they are not offered the opportunity. Just like, “Hey, do you want to go get a Pap smear or some test?”*. *I know that it is also difficult to socialize things such as, for example, emergency contraception, or… Well, birth control methods, right? I suppose because there is this idea that here* [in the shelter] *it is only women, right? So, if they are not with a male partner here, they are not, let´s say, sexually active. But we know that it can also happen between women, right?*”. **Psychologist, migrant shelter, 25 yo, female, Ciudad Juárez**
SRH management skills are associated with SRH utilization	• Women’s agency as a key facilitator to health care utilization	“*For example, I know that in some shelters there are safe spaces for women, girls and adolescents. So, in addition to having a budget so that all shelters implement safe spaces, that they could also implement… So that not only gender-based violence is discussed, right? But also, more information regarding sexual and reproductive health. As we said a while ago, fostering agency, so that women who are in transit, who are waiting in Juárez, can themselves have this capacity to choose, even*”. **Psychologist, NGO, 31 yo, female, Ciudad Juárez**
Instrumental social support modifies the association between time in Mexico and SRH service utilization	• Having instrumental social support is a facilitator of health care utilization • Creating ties with other migrant women or with service providers can reduce the stigma around SRH • Knowledge and SRH literacy as key facilitators to SRH service utilization • Lack of SRH literacy drives the presence of myths and stigma	***“****I remember a woman from Guatemala who cared for another woman’s children so that the other woman, for example, could go to work. And then, on one of her* [the other woman’s] *rest days, she would go to her psychological appointment*”. **Psychologist, international NGO, 31 yo, female, Ciudad Juárez** “*And well, by asking around, I realized that migrants spread the word, and they have their WhatsApp groups. So, they start sharing like, “You can find medicine for free here”, and so they come*”. **Social worker, local decision maker, 40 yo, female, Ciudad Juárez** “*So, giving the information and increasing awareness of the importance of seeking care, but they are also like, “Okay, but who is else is going to go?”. Well, we are inviting all the clients of* [NAME OF NGO]. *And they say, “Well, if five go, I will go, and we will see each other there”. They know each other, maybe there they might start a friendship. That is also what it´s about, right? These spaces allow women to also get to know each other and create networks. I think that is also the plus of providing accompaniment, because you intervene so that get to know each other better, so that they talk, “Ah, look, she is from Honduras”. And then, “Oh yes, the gunshots”. And then, from a plate of food, it stars, right? In other words, meeting each other and being like, “Oh, did you know that eight days ago I met such and such person and we talked, and it was super cool”. I don´t know. It´s also about creating networks between them*”. **Social worker, NGO, 33 yo, female, Mexico City** “*Well, people who have been in Ciudad Juárez for longer have an easier time accessing services because they know more about where to go, or the organizations* [present in Ciudad Juárez]*, how the health system is structured*”*.* **Psychologist, international NGO, 31 yo, female, Ciudad Juárez** “*When I explained to them, well, this is normal in menstruation and this isn´t, many of them had menstrual alterations. But they have not come up in the health assessments because when I ask them “Do you have a normal period? Have you felt anything? Do you get your periods regularly?, and so on. Well, they all said “Yes”. But when I went into more details, many of them had not had their period regularly, some of them had amenorrhea, others had heavy bleedings*”. **Nurse practitioner, Migrant shelter, 28 yo, female, Ciudad Juárez** “*Also these myths, well not myths, but challenges of “Look, she scratches herself a lot here on her* [private] *parts and then she goes and grabs things without washing her hands. She is going to give me this. How am I going to use the bathroom? What if I get infected?*”. **Social worker, Migrant shelter, 24 yo, female, Ciudad Juárez**

1) UITMW: undocumented in-transit migrant women; 2) SRH: sexual and reproductive health

#### Characteristics of interview participants

Participants were mostly women (77.8%), with a median age of 35 years (range 25 to 62) and diverse professional backgrounds, including physicians, nurses, lawyers, psychologists, anthropologists, social workers, education specialists, sociologists, economists, and political scientists. Of the 31 interviews, 19 were conducted with service providers working in migrant shelters, NGOs, international agencies, and public-sector clinics and hospitals. The remaining interviews included four of each: local decision-makers, federal decision-makers, and migration experts. Most participants were based in Ciudad Juárez at the time of the interviews (71.0%), followed by Mexico City (25.8%) and Chihuahua City (3.2%).

#### SRH needs are prevalent among UITMW and increase with longer stays in Mexico

Interviewees echoed survey findings, emphasizing that SRH needs vary based on the migration context such as whether women migrate accompanied or alone, their travel route, and where they come from. Service providers noted that by the time women reach the border, they have often faced multiple adverse events, leading to complex SRH needs.

Survey data showed that women who spent more time in Mexico reported more SRH needs. Local health decision-makers and service providers attributed this to long stretches in border cities like Ciudad Juárez, where women waiting for an opportunity to cross into the U.S. may have more opportunities to recognize and address SRH needs, if no urgent competing priorities arise. However, local and federal decision-makers, as well as service providers from international NGOs, observed that extended stays may be linked to new or additional SRH needs due to poor living conditions, immigration status (e.g., temporal residence and working permit), or changes in UITMW’s migration plans, such as starting a family in Ciudad Juárez while they wait to cross the border. They perceive that women living outside of shelters, with limited access to informal and formal social support, face greater exposure to sexual violence, forced sex work, or drug trafficking, further exacerbating their SRH needs.

#### SRH service utilization is low among UITMW

Even though most women reported at least one SRH need, survey findings indicate that two-thirds did not utilize SRH services, being even lower among women exposed to sexual violence. All interviewees highlighted obstacles that limit UITMW from using SRH services during transit, with fear being a primary deterrent. They perceive that women hesitate to report sexual violence, believing perpetrators—often linked to organized crime—have “eyes everywhere,” even within government institutions. Interviewees also noticed that distrust of authorities, shaped by past abuse from police, migration officials, and healthcare providers, further discourages service use. Interviewees opined that greater trust in government institutions and authorities may facilitate SRH service utilization. Being on the move is yet another impediment, as interviewees perceived that UITMW prioritize the need to keep moving, finding shelter and income sources, and their children’s health over their own SRH needs. Health service providers shared how the lack of care has tangible consequences on the SRH of UITMW. For instance, one physician noted that most pregnant UITMW who access care in one of the public-sector hospitals in Ciudad Juárez do not receive any prenatal care, often presenting with complications and adverse pregnancy outcomes.

All interviewees highlighted how shelters and inter-sector collaborations play a key role in facilitating SRH access. Shelters—where all surveyed participants stayed—provide information, in-house medical staff, and accompaniment to medical appointments, reducing service denial. Although interviewees recognize that shelters are vital in connecting women to health services, they identified challenges in the provision of care stemming from the strict foundational religious values of shelters that object to the provision of birth control, emergency contraception, and abortion care. Faith-based shelter managers did not mention this barrier during the interviews. As for inter-sector collaboration, Ciudad Juárez recently created an inter-agency committee to address the health needs of migrant populations. Several interviewees underscored that this committee has helped reduce healthcare barriers by identifying access gaps and coordinating responses to migrant health needs.

#### SRH management skills are associated with SRH service utilization

Although UITMW encounter multiple barriers to care, SRH management skills were associated with greater service utilization among those who used SRH services. Interviewees reinforced these findings, sharing insights into how these factors might influence SRH service utilization. A social worker from an NGO shared that, based on her experience, UITMW value meeting other women in similar situations because it reduces feelings of isolation, allows them to exchange information on navigating the healthcare system, and even leads to accompanying one another to medical appointments. Interviewees noted that, if given the opportunity, most women can develop the knowledge, resources, and agency to navigate SRH services. Some NGOs have introduced interventions to enhance women’s SRH management skills, including building awareness, confidence, and the ability to seek care independently within the public health system.

#### Instrumental social support modifies the association between time in Mexico and SRH service utilization

The interviews provided context on why a lack of instrumental social support increases the odds of seeking healthcare the longer UITMW stay in Mexico. Several service providers perceive that social support facilitates timely healthcare use by enabling childcare, sharing health service information, fostering connections with other women, and growing awareness of health rights. Interviewees also described how social ties may foster a sense of belonging and safety, potentially reducing stigma and shame around SRH and making it easier for women to recognize and seek care. Additionally, all interviewees perceived that limited SRH literacy—more common among women with primary education—is a barrier to recognizing SRH needs, seeking care, and dispelling myths and stigma around SRH.

In summary, the qualitative findings show that the confluence of individual characteristics, experiences in the migration journey, and the resources available in the in-transit communities affect UITMW’s ability to access and utilize SRH services. Along with having individual SRH management skills to overcome barriers, ensuring the availability of instrumental support, the presence of shelters, and interagency collaborations in the local communities emerged as key facilitators of SRH service utilization for UITMW in Mexico.

## Discussion

Increasingly restrictive immigration policies are forcing UITMW to spend more time in Mexico, where they face multiple hardships affecting their SRH and access to care. Our study found that despite a high prevalence of self-reported SRH needs, which increase with longer stays in Mexico, there is low utilization of SRH services, indicating barriers to care. Additionally, we found that utilization of SRH services is associated with instrumental social support and SRH management skills.

A key contribution of our study is the link between prolonged stays in Mexico and rising SRH needs. While limited evidence exists on the mechanisms driving this increase, interview data suggests that health and migration policies in Mexico fail to address the needs of UITMW who find themselves in a protracted situation while waiting for an opportunity to cross into the U.S. [35]. Prolonged stays may not necessarily lead to improved living conditions, increased access to immigration documents, stable income, reduced exposure to sexual violence, or access to health care, all of which impact UITMW’s SRH [53].

Despite the high prevalence of SRH needs and legal protections ensuring UITMW’s right to health services in Mexico, we found low utilization of health services among UITMW, aligning with findings from previous studies on in-transit migrant populations [28, 31]. Our findings also identified faith-based shelters as barriers to SRH care. While shelters are often recognized as key providers of health services for undocumented in-transit migrants in Mexico [31, 34], the fact that most are managed by the Catholic Church creates tensions between religious pro-life values and the SRH needs of UITMW. Similarly, Llanes-Díaz et al. found that the religiosity of shelters act as a barrier to the provision of comprehensive SRH services for this population [26].

Furthermore, our findings show that among women without instrumental social support, SRH service utilization was more common among those who had spent longer periods in Mexico. While this pattern does not imply a causal relationship, it may reflect that women without social support, compared to those who have this support, face greater challenges accessing care early on. Our qualitative data suggest that limited awareness of health rights and available services, fewer peer networks, and the absence of trusted childcare may delay healthcare-seeking among this group. In contrast, women who reported having instrumental social support tended to have higher education levels and greater SRH knowledge, which may help explain the lack of an association between length of stay and service use among them. As Hernández-Plaza et al. note, informal social networks often serve as primary sources of information and support for migrants [37]. For women traveling with children—who represented the majority of our sample—limited childcare options in shelters may further constrain their ability to seek care. This aligns with findings from a Canadian study that identified caregiving responsibilities as a barrier to SRH service use among migrants with limited social support [54].

While in our study SRH management skills were positively associated with SRH service utilization, and interviewees identified knowledge, information, and agency as key facilitators for accessing care, these factors alone may be insufficient to guarantee access when women lack trust in government institutions and face highly violent contexts. A scoping review conducted by Garbett and colleagues examined the choice paradox faced by UITMW in Mexico and found that UITMW’s SRH decisions are constrained by the violence they experience in Mexico, violations of their SRH and human rights, and organizational and structural barriers that limit their ability to access SRH care [24].

Overall, our findings suggest a high prevalence of SRH needs and gaps in SRH service provision and utilization among UITMW transiting through Mexico. They point to the need for strengthening inter-sector collaboration, expanding formal and informal social support networks, and enhancing SRH management skills among migrant women as strategies to reduce barriers to care. These factors may help ensure more timely and responsive care for women navigating extended and precarious stays in Mexico.

As women spend increasing amounts of time in Mexico, often without regularizing their migration status to preserve eligibility for US asylum, many remain in shelters or informal settlements under precarious conditions. Current programs from NGOs, shelters, and government actors tend to focus on urgent, short-term needs. However, the extended nature of migration journeys calls for a paradigm shift: policies and interventions must begin to address the evolving needs of populations in protracted transit, including their SRH needs. In this context, improving timely access to care may reduce health complications, lower treatment costs, and improve their well-being [55, 56].

Continued research is essential to better understand the SRH vulnerabilities that emerge during these prolonged stays. Future studies should go beyond traditional healthcare access-utilization frameworks to examine how structural, legal, and social dynamics shape migrant women’s ability to access care and exercise their rights. Specifically, more research is warranted on how different forms of social support and caregiving responsibilities affect healthcare decisions, especially for women traveling with children, under conditions of constrained choice and limited agency.

This study contributes to understanding the SRH needs and service utilization of UITMW by integrating perspectives from both migrant women and a diverse range of stakeholders. Nonetheless, several limitations should be considered.

The survey sample was limited to women staying in shelters, which may have led to an underestimation of SRH needs and barriers experienced by those living outside the shelter system. Additionally, we lacked information on non-respondents, which limits our ability to assess potential differences between those who participated and those who declined, and may introduce selection bias. Despite efforts to build trust through trauma-informed practices, the use of an interviewer-administered format and the inclusion of questions about current and past SRH needs and service utilization may have introduced recall or social desirability bias, potentially influencing responses to sensitive topics. Furthermore, small sample sizes contributed to wide 95% CIs, which should be considered when interpreting associations. In addition, the categorization of time spent in Mexico, based on official surveys [7], may no longer reflect current migration patterns and warrants re-evaluation. Finally, the survey was conducted in 2021, during Mexico’s COVID-19 “stay-at-home” orders, whereas interviews took place in 2023 after restrictions were lifted. This timing difference complicates comparisons between survey and interview responses, as healthcare services and migrant shelters operated under different conditions. For example, during COVID-19, some shelters enforced stricter exit and reentry policies, which may have affected UITMW’s access to SRH services. However, key findings from the survey were corroborated by interviewees, suggesting persistent SRH needs and barriers to care.

The qualitative study did not include direct interviews with UITMW. Ethical concerns about re-traumatization and limited access to shelters, some of which cited concerns about extractive research, prevented their inclusion. Although this limits direct comparison across the mixed-methods study, all interviewed stakeholders had extensive experience working directly with UITMW or in policy design. We believe that the strong consensus among participants and the diversity of perspectives included lend credibility to the qualitative findings and enhance the interpretation of survey results.

## Conclusions

This is one of the few studies that explores the role of time and instrumental social support in shaping SRH needs and the use of SRH services among UITMW. The study findings show that longer time spent in Mexico is associated with increased self-reported SRH needs, but that these needs do not translate into more use of SRH services. Women without instrumental social support may face greater challenges in utilizing SRH services, potentially delaying their contact with care, particularly for time-sensitive services, such as those related to sexual violence. Consistent with the experience of migrant women in other countries, the results suggest persistent gaps in SRH care for this population. Understanding the additional barriers to SRH care faced by UITMW staying for longer in transit countries and how particular changes in the migration dynamics influence health needs and utilization of health services may inform improvements in the health system’s response to these needs and the SRH outcomes of UITMW.

## Supplementary Information

Additional File 1. Sexual and reproductive health needs and service utilization variables: Survey items and coding for analyses. This file provides the original survey questions used to construct the SRH needs and service utilization variables, along with the corresponding coding schemes applied in the quantitative analysis.

Additional File 2. Absolute difference in the probability (ADP) of using SRH services by time spent in Mexico and perceived availability of instrumental social support. This document presents the results of the effect measure modification analysis on the additive scale, including both unadjusted and adjusted absolute differences in probability for key subgroups.

Additional file 3. This file includes the Consolidated Criteria for Reporting Qualitative Studies (COREQ) checklist used to ensure transparent and comprehensive reporting of the qualitative findings in this study.

Additional File 4. Interview guides for service providers, decision-makers, and migration experts. This file contains the semi-structured interview guides used to conduct qualitative interviews with service providers, local and federal decision-makers, and experts working with migrant populations. The guides are available in English and Spanish.

Additional File 5. Qualitative codebook. This file contains the finalized qualitative codebook, including both a priori and inductively developed codes used in the thematic analysis of interview data.

## Data Availability

The quantitative dataset used in this study is publicly available at https://doi.org/10.7910/DVN/GVEVGN [44]. Access to the dataset is subject to the terms and conditions specified by the Population Council, as outlined in the link provided. The qualitative data supporting this study’s findings are not publicly available due to confidentiality concerns and ongoing related publications. However, the data may be made available from the first author (SL) upon reasonable request, subject to approval and in accordance with ethical guidelines.

## References

[1] International Organization for Migration. World Migration Report 2022. Geneva: IOM; 2021.

[2] International Organization for Migration. Glossary on Migration. Geneva: International Organization for Migration; 2019.

[3] Unidad de Política Migratoria. Boletines Estadísticos. Unidad de Política Migratoria, Gobierno de México. https://portales.segob.gob.mx/es/PoliticaMigratoria/Boletin_MyH. Accessed 6 Dec 2023.

[4] Organización Internacional para las Migraciones. Perfil Migratorio de México. Boletín Anual 2022. Mexico City: Organización Internacional para las Migraciones; 2023.

[5] Women´s Refugee Commission, Instituto para las Mujeres en la Migración. Stuck in uncertainty and exposed to violence: the impact of US and Mexican migration policies on women seeking protection in 2021. Mexico City: WRC & IMUMI; 2022.

[6] International Organization for Migration. Migration within the Americas. Missing Migrants Project. 2024. https://missingmigrants.iom.int/region/americas?region_incident=4076&route=All&incident_date%5Bmin%5D=&incident_date%5Bmax%5D=. Accessed 25 Mar 2024.

[7] El Colegio de la Frontera Norte. Encuestas sobre migración en las fronteras norte y sur de México. EMIF Norte Sur. 2024. https://www.colef.mx/emif/. Accessed 11 Apr 2024.

[8] United Nations Human Rights. Human rights in transit and at international borders. OHCHR and migration. 2022. https://www.ohchr.org/en/migration/human-rights-transit-and-international-borders#:~:text=Many%20migrants%20in%20transit%20are,never%20reach%20their%20intended%20destination. Accessed 14 May 2024.

[9] Médicos Sin Fronteras. Sin Salida. La Crisis Humanitaria de la Población Migrante y Solicitante de Asilo Atrapada entre Estados Unidos, México y el Triángulo Norte de Centroamérica (TNCA). Mexico: Médicos Sin Fronteras; 2020.

[10] Infante C, Leyva-Flores R, Gutierrez JP, Quintino-Perez F, Torres-Robles CA, Gomez-Zaldívar M. Rape, transactional sex and related factors among migrants in transit through Mexico to the USA. Cult Health Sex. 2020;22:1145–60. 31682779 10.1080/13691058.2019.1662088

[11] Infante C, Silván R, Caballero M, Campero L. Sexualidad del migrante: experiencias y derechos sexuales de centroamericanos en tránsito a los Estados Unidos. Salud Publica Mex. 2013;55:58. 23918058

[12] Ramage K, Stirling-Cameron E, Ramos NE, Martinez SanRoman I, Bojorquez I, Spata A, et al. “When you leave your country, this is what you’re in for”: experiences of structural, legal, and gender-based violence among asylum-seeking women at the Mexico-U.S. border. BMC Public Health. 2023;23:1699. 10.1186/s12889-023-16538-2 PMC10474729 37659997

[13] International Planned Parenthood Federation. IMAP Statement on sexual and reproductive health services in humanitarian settings. London: IPPF; 2018.

[14] Starrs AM, Ezeh AC, Barker G, Basu A, Bertrand JT, Blum R, et al. Accelerate progress—sexual and reproductive health and rights for all: report of the Guttmacher– Lancet Commission. The Lancet. 2018;391:2642–92. 10.1016/S0140-6736(18)30293-9 29753597

[15] Leyva-Flores R, Infante C, Servan-Mori E, Quintino-Pérez F, Silverman-Retana O. HIV prevalence among central American migrants in transit through Mexico to the USA, 2009–2013. J Immigrant Minority Health. 2016;18:1482–8. 10.1007/s10903-015-0268-z 26359004

[16] Quintino-Pérez F, Montoya A, Gómez-López D, Vázquez-Aguilar E, Cortés Alcalá R, Leyva-Flores R. Dinámica de movilidad y salud de mujeres migrantes en México, en el contexto de la pandemia Covid-19, 2021–2022. Salud Publica Mex. 2024;66:137–49. 39977043 10.21149/14812

[17] Leyva-Flores R, Aracena-Genao B, Bustamante ND, Bojorquez I, Cortés-Alcalá R, Gómez-López D, et al. Ten-year hospitalization trends in Mexico: examining the profile of national and transient and migrants. Front Public Health. 2023;10:1060861. 36761333 10.3389/fpubh.2022.1060861 PMC9905618

[18] Letona P, Felker-Kantor E, Wheeler J. Sexual and reproductive health of migrant women and girls from the Northern Triangle of Central America. Panam Salud Publica. 2023. 10.26633/RPSP.2023.59. 10.26633/RPSP.2023.59 PMC10003450 36909804

[19] Vázquez-Quesada L, Peña J, Vieitez-Martínez I. Necesidades y atención en salud sexual y reproductiva de mujeres migrantes en México. Un estudio desde Ciudad Juárez, Chihuahua. Volumen I. Mexico: Population Council & El Colegio de la Frontera Norte; 2021.

[20] International Organization for Migration. Displacement Tracking Matrix. Flow monitoring of migrants in Tapachula and Tenosique, Mexico. Round 1. Health and Migration. Mexico: IOM; 2022.

[21] Cámara de Diputados. Ley General de Salud. 2022.

[22] Gobierno de México. Ley General de Población. 1974.

[23] Secretaría de Salud. Plan Integral de Atención a la Salud de la Población Migrante. 2020.

[24] Garbett A, De Oliveira Tavares NC, Riggirozzi P, Neal S. The paradox of choice in the sexual and reproductive health and rights challenges of south-south migrant girls and women in Central America and Mexico: a scoping review of the literature. Journal of Migration and Health. 2023;7: 100143. 36568827 10.1016/j.jmh.2022.100143 PMC9768374

[25] Quesada LV, Larrea-Schiavon S, Marin TT, García GBM, Basurto-Alcalde E, Ochoa B, et al. Mujeres migrantes en Tapachula, Mexico: barreras y facilitadores para el acceso a la salud sexual y reproductiva en 2020—Resumen de la investigación. Population Council; 2021.

[26] Llanes-Díaz N, Bojórquez-Chapela I, Odgers-Ortiz O. Oferta de servicios de salud sexual y reproductiva a personas migrantes centroamericanas en Tijuana. Panam Salud Publica. 2023;47:1–9. 10.26633/RPSP.2023.56 PMC9989548 36895679

[27] Vázquez-Quesada L, Peña J, Vieitez-Martinez I. Necesidades y atención en salud sexual y reproductiva de mujeres migrantes en México. Un estudio desde Ciudad Juárez, Chihuahua. Volumen II. Mexico City: Population Council & El Colegio de la Frontera Norte; 2021.

[28] Leyva R, Infante C, Quintino-Perez F, Domínguez J, Santos E. Migración y redes sociales para la salud sexual y reproductiva: experiencias del Programa Conjunto de Migrantes en Tránsito por México. Mexico: Instituto Nacional de Salud Pública; 2016.

[29] Larrea-Schiavon S, Vázquez-Quesada L, Basurto-Alcalde E, Polgovsky N, Vieitez Martínez I. Atención de la salud sexual y reproductiva de mujeres migrantes: Un mapeo de actores de la sociedad civil en México. Mexico City: Population Council; 2021.

[30] Peña J, Vázquez-Quesada L, Vieitez-Martínez I. Necesidades de Atención en Salud Sexual y Reproductiva de Mujeres Migrantes en México. Un estudio desde Ciudad Juárez, Chihuahua. Volumen III. Mexico: Population Council & El Colegio de la Frontera Norte; 2022.

[31] Leyva-Flores R, Infante C, Quintino-Perez F. Migrantes en tránsito por México: situación de salud, riesgos y acceso a servicios de salud. Mexico: Instituto Nacional de Salud Pública; 2016.

[32] Leyva-Flores R, Infante C, Serván-Mori E, Quintino-Perez F, Silverman-Retana O. Acceso a Servicios de Salud para los Migrantes Centroamericanos en Tránsito por México. Policy Brief. Mexico: CANAMID; 2015.

[33] Piérola MD, Rodríguez M. Migrants in Latin America: disparities in health status and in access to healthcare. Washington, D.C.: Inter-American Development Bank; 2020.

[34] Infante C, Vieitez-Martinez I, Rodríguez-Chávez C, Nápoles G, Larrea-Schiavon S, Bojorquez I. Access to health care for migrants along the Mexico-United states border: applying a framework to assess barriers to care in Mexico. Frontiers in Public Health. 2022;10. 10.3389/fpubh.2022.921417 PMC9330619 35910916

[35] Bojórquez-Chapela I, Infante-Xibille C, Rodríguez-Chávez C, Larrea-Schiavon S, Vieitez-Martínez I. Atención en salud de Covid-19 para migrantes en México: análisis desde la problematización de la política pública. Salud Publica Mex. 2023;:1–7. 10.21149/14836 39977041

[36] Cohen S, Wills TA. Stress, social support, and the buffering hypothesis. Psychol Bull. 1985;98:310–57. 3901065

[37] Hernandez-Plaza S, Alonso-Morillejo E, Pozo-Munoz C. Social support interventions in migrant populations. Br J Soc Work. 2005;36:1151–69.

[38] Vu M, Besera G, Ta D, Escoffery C, Kandula NR, Srivanjarean Y, et al. System-level factors influencing refugee women’s access and utilization of sexual and reproductive health services: a qualitative study of providers’ perspectives. Front Glob Womens Health. 2022;3:1048700. 36589147 10.3389/fgwh.2022.1048700 PMC9794861

[39] Aibangbee M, Micheal S, Mapedzahama V, Liamputtong P, Pithavadian R, Hossain Z, et al. Migrant and refugee youth’s sexual and reproductive health and rights: a scoping review to inform policies and programs. Int J Public Health. 2023;68:1605801. 37342678 10.3389/ijph.2023.1605801 PMC10278890

[40] Akresh IR. Health service utilization among immigrants to the United States. Popul Res Policy Rev. 2009;28:795–815.

[41] Greene JC, Caracelli VJ, Graham WF. Toward a conceptual framework for mixed-method evaluation designs. Educ Eval Policy Anal. 1989;11:255–74.

[42] Andersen RM. Revisiting the behavioral model and access to medical care: Does it Matter? J Health Soc Behav. 1995;36:1. 7738325

[43] Unidad de Política Migratoria. Diagnóstico de la Movilidad Humana en Chihuahua. Mexico: Unidad de Política Migratoria; 2022.

[44] Vázquez-Quesada, Lucía; Vieitez-Martínez, Isabel; Peña, Jesús, 2025, "Sexual and reproductive health of migrant women in Ciudad Juárez, Mexico 2021", 10.7910/DVN/GVEVGN, Harvard Dataverse, V1.

[45] Schlegel EC, Pickler RH, Tate JA, Williams KP, Smith LH. The EMeRGE theory of emerging adult-aged women’s sexual and reproductive health self-management: a grounded theory study. J Adv Nurs. 2023;80:510–25. 37533185 10.1111/jan.15814 PMC10834842

[46] World Health Organization. Self-care interventions for sexual and reproductive health. Sexual and Reproductive Health and Research. 2023. https://www.who.int/teams/sexual-and-reproductive-health-and-research-%28srh%29/areas-of-work/self-care-interventions?utm_source=chatgpt.com.

[47] Knol MJ, VanderWeele TJ. Recommendations for presenting analyses of effect modification and interaction. Int J Epidemiol. 2012;41:514–20. 22253321 10.1093/ije/dyr218 PMC3324457

[48] StataCopr LLC. Stata BE.

[49] Tong A, Sainsbury P, Craig J. Consolidated criteria for reporting qualitative research (COREQ): a 32-item checklist for interviews and focus groups. Int J Qual Health Care. 2007;19:349–57. 17872937 10.1093/intqhc/mzm042

[50] Quinn PM. Qualitative research and evaluation methods. 4th ed. United States: SAGE Publications; 2015.

[51] VERBI GmbH. MAXQDA.

[52] Klingberg S, Stalmeijer RE, Varpio L. Using framework analysis methods for qualitative research: AMEE Guide No. 164. Medical Teacher. 2023;:1–8. 10.1080/0142159X.2023.2259073 37734451

[53] Williams DR, Costa MV, Odunlami AO, Mohammed SA. Moving upstream: how interventions that address the social determinants of health can improve health and reduce disparities. J Public Health Manag Pract. 2008;14:S8-17. 18843244 10.1097/01.PHH.0000338382.36695.42 PMC3431152

[54] Neufeld A, Harrison MJ, Stewart MJ, Hughes KD, Spitzer D. Immigrant women: making connections to community resources for support in family caregiving. Qual Health Res. 2002;12:751–68. 12109721 10.1177/104973230201200603

[55] Christopher AS, McCormick D, Woolhandler S, Himmelstein DU, Bor DH, Wilper AP. Access to care and chronic disease outcomes among Medicaid-insured persons versus the uninsured. Am J Public Health. 2016;106:63–9. 26562119 10.2105/AJPH.2015.302925 PMC4695932

[56] Hearld LR, Alexander JA. Patient-centered care and emergency department utilization: a path analysis of the mediating effects of care coordination and delays in care. Med Care Res Rev. 2012;69:560–80. 22813721 10.1177/1077558712453618

