# Supplementary material for: Sexual and reproductive health of in-transit migrant women en route to the United States: a mixed-methods study in Ciudad Juárez, Mexico
Source: BMC Glob Public Health. 2025 Jul 7;3:60. doi: 10.1186/s44263-025-00180-8 (PMC12235796; doi:10.1186/s44263-025-00180-8)
Supplement: Supplementary file 1 — Additional File 1. Sexual and reproductive health needs and service utilization variables: Survey items and coding for analyses. This file provides the original survey questions used to construct the SRH needs and service utilization variables, along with the corresponding coding schemes applied in the quantitative analysis. [file 44263_2025_180_MOESM1_ESM.docx]

| **Additional File 1. Sexual and reproductive health needs and service utilization variables: Survey items and coding for analyses.** | | |
| --- | --- | --- |
| **Variables** | **Corresponding survey item** | **Coded for analyses as** |
| Menstrual health and management | During your transit through Mexico: 1) Did you have menstrual pains that prevented you from carrying out daily activities (e.g. very strong cramps)? 2) Did you have irregular menstrual cycles?; 3) Were you able to keep your genitals clean every day?; 4) Had clean, unused cloths to wipe yourself?; 5) Had enough clean, unused menstrual pads or tampons?; and, 6) Were you able to change your menstrual pads or tampons as needed? | 0 = Always able to follow these practices during their trip to Mexico, never experienced severe menstrual pains, and never experienced irregular cycles.  1 = Never or only sometimes able to follow one or more of these practices, experienced severe menstrual pains, or experienced irregular cycles during their trip to Mexico. |
| Reproductive tract infections-related symptoms | During your transit through Mexico, have you had any of the following symptoms in your genitals? Bleeding other than menstruation, warts, pain, itching, hives, problems urinating, abnormal vaginal discharge, or ulcers. | 0 = Did not experience any of these symptoms.  1 = Experienced one or more of these symptoms. |
| Sexual violence | During the time you have been in Mexico, have you experienced inappropriate touchings (inappropriate fondles)? | 0 = No  1 = Yes |
| Contraception | During your transit through Mexico, did you use any contraceptive method to avoid getting pregnant during the trip? | 0 = No  1 = Yes |
| Pregnancy | During your transit through Mexico, have you ever been pregnant? | 0 = No  1 = Yes |
| SRH needs binary variable | Variable that indicates if UITMW presented at least one of the previous SRH needs. | 0 = Did not report any of the previous SRH needs.  1 = Reported at least one of the previous SRH needs. |
| SRH needs continuous variable | Number of SRH needs experienced. | Continuous variable. Range from 0 to 12 needs. |
| SRH service utilization | We created a binary variable for SRH service utilization by combining the following survey questions. 1) Did you receive medical care for the RTI-related symptoms?; 2) Did you receive medical care for any symptom experienced after a sexual intercourse?; 3) Did you receive medical care at any point during pregnancy?; and, 4) Were you able to access menstrual pain medications? | 0 = No  1 = Yes to at least one SRH service |
