# Supplementary material for: Sexual and reproductive health of in-transit migrant women en route to the United States: a mixed-methods study in Ciudad Juárez, Mexico
Source: BMC Glob Public Health. 2025 Jul 7;3:60. doi: 10.1186/s44263-025-00180-8 (PMC12235796; doi:10.1186/s44263-025-00180-8)
Supplement: Supplementary file 2 — Additional File 2. Absolute difference in the probability (ADP) of using SRH services by time spent in Mexico and perceived availability of instrumental social support. This document presents the results of the effect measure modification analysis on the additive scale, including both unadjusted and adjusted absolute differences in probability for key subgroups. [file 44263_2025_180_MOESM2_ESM.docx]

| **Additional File 2. Absolute difference in the probability (ADP) of using SRH services by time spent in Mexico and perceived availability of instrumental social support. Results of Effect Measure Modification analysis on the additive scale.** | | | | |
| --- | --- | --- | --- | --- |
| **Variables** | **Total sample** | | **Perceived availability of instrumental social support** | |
|  | **Unadjusted** | **Adjusted** | **Yes** | **No** |
|  | **Model 1**  cADP (95% CI)  (Total sample) | **Model 2**  aADP (95% CI)  (Total sample) | **Model 3**  aADP (95% CI) | **Model 4**  aADP (95% CI) |
|  | **n = 252** | **n = 195** | **n = 127** | **n = 68** |
| Time in Mexico |  |  |  |  |
| ≤ 15 days | REF | REF | REF | REF |
| 16 – 30 days | -0.08 (-0.21 – 0.05) | -0.05 (-0.21 – 0.10) | -0.02 (-0.21 – 0.16) | -0.13 (-0.46 – 0.20) |
| >30 days | **0.14 (0.005 – 0.28)*** | 0.13 (-0.03 – 0.30) | 0.01 (-0.21 – 0.23) | **0.35 (0.06 – 0.64)*** |
| **Predisposing factors** |  |  |  |  |
| Educational level |  |  |  |  |
| Basic education | - | REF | REF | REF |
| >Basic education | - | -0.03 (-0.18 – 0.12) | 0.003 (-0.18 – 0.19) | -0.02 (-0.32 – 0.29) |
| Marital status |  |  |  |  |
| Single and not currently living with a partner | - | REF | REF | REF |
| Cohabitating or married but not currently living with partner | - | 0.07 (-0.12 – 0.26) | 0.03 (-0.20 – 0.27) | 0.26 (-0.10 – 0.62) |
| Cohabitating or married and currently living with partner | - | **0.14 (-0.01 – 0.29)^** | 0.08 (-0.10 – 0.27) | 0.21 (-0.05 – 0.47) |
| Knowledge about rights and/or where to get care |  |  |  |  |
| Does not know about rights | - | REF | REF | REF |
| Knows about rights | - | -0.01 (-0.16 – 0.15) | 0.05 (-0.16 – 0.25) | -0.10 (-0.36 – 0.16) |
| **Enabling factors** |  |  |  |  |
| Received information about SRH and/or SRH services in Mexico |  |  |  |  |
| No | - | REF | REF | REF |
| Yes | - | 0.06 (-0.16 – 0.28) | 0.05 (-0.24 – 0.35) | 0.07 (-0.30 – 0.44) |
| Perceived availability of instrumental social support |  |  |  |  |
| No | - | REF | - | - |
| Yes | - | -0.01 (-0.15 – 0.13) | - | - |
| Perception of difficulty of solving an SRH need | - | **-0.05 (-0.08 – 0.02)*** | **-0.07 (-0.11 – -0.03)*** | -0.03 (-0.09 – 0.04) |
| Perceived ability to manage one´s SRH needs |  |  |  |  |
| No | - | REF | REF | REF |
| Yes | - | **0.15 (0.01 – 0.29)*** | **0.18 (0.001 – 0.37)*** | 0.15 (-0.11 – 0.40) |
| Number of adverse events experienced during transit |  |  |  |  |
| <5 risks | - | REF | REF | REF |
| 5-8 risks | - | 0.00 (-0.15 – 0.15) | 0.03 (-0.16 – 0.23) | -0.01 (-0.29 – 0.27) |
| 9-12 risks | - | -0.01 (-0.24 – 0.22) | 0.02 (-0.26 – 0.31) | 0.07 (-0.36 – 0.51) |
| Number of perceived SRH needs while in transit | - | 0.01 (-0.01 – 0.03) | **0.02 (-0.004 – 0.05)^** | -0.03 (-0.07 – 0.01) |
| **Variance Inflation Factor (VIF)** | - | 1.25 | 1.27 | 1.46 |
| **Note:** *p-value ≤0.05; ^p-value ≤0.10 | | | | |
