# Supplementary material for: Sexual and reproductive health of in-transit migrant women en route to the United States: a mixed-methods study in Ciudad Juárez, Mexico
Source: BMC Glob Public Health. 2025 Jul 7;3:60. doi: 10.1186/s44263-025-00180-8 (PMC12235796; doi:10.1186/s44263-025-00180-8)
Supplement: Supplementary file 5 — Additional File 5. Qualitative codebook. This file contains the finalized qualitative codebook, including both a priori and inductively developed codes used in the thematic analysis of interview data. [file 44263_2025_180_MOESM5_ESM.docx]

**Additional File 5. Codebook.**

1. ***Participants background and profile***
2. **Professional background**

- Includes information about academic training, work with migrants in general, and work with migrant women specifically.
- Subcode: SRH (work on SRH)
- Examples: Psychologist, working with migrant women since 2020. Main work revolves around offering psychosocial services to the population.

1. **Personal background**

- Includes information about identities of participants interviewed, personal perspectives (e.g., feminist), and other relevant information on participants mental maps that influences how they approach the work they do.
- Example: Participant self-identifies as a feminist researcher. Believes in agency of migrant women and the power and importance of community-level organization.

1. **Organization/Institution background**

- Includes information about mission, focus, programs, target population, and location where the organization/institution does work, as well as services offered by the organization.
- Example: International organization working in Mexico since 2020 and in Ciudad Juárez since 2022. Specializes in preventing and addressing gender-based violence. Programs implemented for migrant women have focus on providing resources to increase their financial stability.

*SEXUAL AND REPRODUCTIVE HEALTH ACCESS AND UTILIZATION SYSTEM (MICRO-SYSTEM)*

1. ***Migration process and profile***
2. **Context of emigration**

- Includes information about context in the countries of origin of migrant women.
- Includes information about reasons why migrant women decide or are forced to leave their countries of origin.
- Example: The health systems of some countries in Central America have not been capable of responding to the health needs of their population. High levels of community violence in some countries.

1. **Context of in-transit migration**

- Includes information about the context faced by women during their transit in Mexico, or other countries they crossed to arrive to Mexico.
- Includes information about routes taken and means of transportation used.
- Includes information about the risks migrant women are exposed to while transiting through Mexico or while in Ciudad Juárez.
- Includes information about the types of violence migrant women are exposed to while transiting through Mexico or while in Ciudad Juárez.
- Includes information about coping mechanisms implemented during transit.
- Examples: Women mostly migrate using coyotes and staying in houses or rented rooms. They do not usually use migrant shelters during transit. Once in Ciudad Juárez, it is more common for them to use migrant shelters, especially if they are survivors of gender-based violence.
- **Subcodes**: Transit; Ciudad Juárez

1. **Profile and trends**

- Include information on the sociodemographic characteristics of migrant women, as well as if and how these profiles have changed in recent years.
- Examples: Before 2019, the migration profiles seen were mostly men. After 2019, there was a change with more families and women migrating, as well as people migrating in caravans.

1. ***Health and other needs***
2. **Health needs**

- Includes information on the main health needs of migrant women while in transit and while in Ciudad Juárez.
- Examples: respiratory diseases, dermatosis, etc…
- **Subcodes**: Transit; Ciudad Juárez

1. **SRH needs**

- Includes information on the SRH needs of migrant women while in transit and while in Ciudad Juárez.
- SRH needs include: sexual violence, other types of gender-based violence, sexual health needs, and reproductive health needs.
- Examples: genitourinary infections and STDs, pregnancy, etc…
- **Subcodes**: Transit; Ciudad Juárez

1. **Other needs**

- Includes information about other non-health related needs of migrant women during transit or while in Ciudad Juárez.
- Examples: housing, education, work, etc
- **Subcodes**: Transit; Ciudad Juárez

1. **Needs changes**

- Includes any information about changes in health needs based: 1) stage of the trip (e.g., country of origin, transit, Ciudad Juárez), or 2) time in Mexico.
- Example: Needs are different based on the stage of the migration trip. During transit they are more exposed to health needs related to sexual violence. Once in Ciudad Juárez, they experience health needs that are related to overcrowded spaces and lack of access to water and sanitation.
- **Subcodes:** Stage; Time

1. **Health services***

- Includes information on where women access health services. What types of services women use the most.

1. ***Barriers and facilitators to care at the individual level***
2. **Barriers and facilitators to care at the individual level**

- Includes information on migrant women regarding:
  - Health literacy, health beliefs, trust and expectations on health needs and health personnel of institutions or organizations, and personal priorities.
  - Personal and social values, culture, autonomy, knowledge, and social networks that facilitate or hinder them from seeking health services.
  - Their living environments, mobility, formal networks, family-related responsibilities that can either facilitate or hinder their ability to reach health services.
  - Income, assets, social capital, and access to health insurance that can either facilitate or hinder their ability to pay and use health services.
  - Agency, information, adherence, caregiver support, and mobility and length of stay that can either facilitate or hinder their ability to engage with health systems.
- Examples: Health is not a priority for migrant women.
- **Subcodes:** facilitator; barrier

1. **Barriers and facilitators to care at the institutional level**

- Includes information on health services provided by governmental institutions or CSOs regarding:
  - Transparency, outreach, information, and screening.
  - Professional values, norms, culture, and gender of providers that either facilitate or hinders women from seeking health services there.
  - Geographic location, accommodation, hours of opening, appointment mechanisms, knowledge, and operationalization of rights that can either facilitate or hinder women´s ability to reach health services.
  - Direct and indirect costs of health services, opportunity costs, and resources available for migrants that can either facilitate or hinder women´s ability to use health services.
  - Technical and interpersonal quality, adequacy of services, referral system, and interpreters which can either facilitate or hinder women´s ability to engage with health systems.
- Example: Government health services implement outreach activities in shelters to provide primary health services to the migrant populations (coding system: approachability + outreach + facilitator).
- **Subcodes:** facilitator; barrier

*HEALTH SYSTEM AND COMMUNITY DRIVERS (MESO-SYSTEM)*

1. **Local policies**

- Includes information on local policies regarding migration and health that are impacting access and use of health services by migrant women.
- Example: Chihuahua is a very conservative state. Abortion care is only available to women if the causal for the abortion is sexual violence (coding system: local policies + barrier).
- **Subcodes:** facilitator; barrier

1. **Programs**

- Includes information on local programs being implemented to respond to the health needs of migrant women.
- Example: One of the PHC centers in Ciudad Juárez was specifically implemented and designed to increase access to PHC services for migrant populations.

1. **Religion***

- Information about how religion or being a religious space hinders or facilitates access to SRH services.

1. **Academia***

- Information around academia and research and how it relates to the topic of migration and health. How academia can positively or negatively impact migrant and other underserved populations.

1. **Local decision-making processes**

- Includes information on how decisions are being made at the local level, as well as who is involved in these decisions.
- Example: Decisions at the local level are mostly based on the political agenda of local decision-makers (coding system: local decision-making processes + barrier).
- **Subcodes:** facilitator; barriers

1. **Communication and collaboration practices**

- Includes information on local communication and collaboration practices that are being implemented at the local level to respond to the health needs of migrant populations.
- Includes information on specifics of this communication and collaboration practices that are either considered as best practices or areas of opportunities.
- Example: organizations and local government institutions have implemented healthcare networks to respond to the health needs of migrant populations (coding system: communication and collaboration practices + best practices).

1. **Sustainability***

- Information about the sustainability of projects/programs or organizations.

1. **Trust in organization/institutions***

- Information about relationships or perceptions of trusts in organizations and/or gov institutions by other organizations.

1. **Local context**

- Includes relevant information about the local context of Ciudad Juárez that is either facilitating or hindering access to health services for migrant women and/or impacting women´s health.
- Includes relevant information on community perceptions of migrant populations in Ciudad Juárez.
- Example: Ciudad Juárez has high levels of gender-based violence and is a known route for human trafficking (coding system: local context + barrier).
- **Subcodes:** facilitator; barrier

*POLITICAL, ECONOMICAL, AND SOCIAL DRIVERS (MACRO-SYSTEM)*

1. **National context**

- Includes relevant information about the national context that is either facilitating or hindering access to health services for migrant women and/or impacting women´s health.
- Example: Political polarization is affecting the implementation of federal programs related to migration and health at the local level (coding system: national context + barrier).
- **Subcodes:** facilitator; barrier

1. **Political drivers**

- Includes information on political factors that impact the provision of health services to migrant populations in Mexico.
- Includes information on national public policies that either facilitate or hinder access and use of health services for migrant women.
- Includes information on federal programs that are being implemented to facilitate access to health services for migrant women.
- Example: The United States dictate migration policies that directly affects how Mexico responds to the migration flows (political drivers + barrier).
- **Subcodes:** facilitator; barrier

1. **Federal decision-making processes**

- Includes information on how decisions are being made at the federal level, as well as who is involved in these decisions.
- Example: Migrant communities are not part of the decision-making process (coding system: federal decision-making process + barrier).
- **Subcodes:** facilitator; barrier

1. **Implementation mechanisms**

- Includes information on if and how the federal programs and instructions are being implemented at the local level.
- Includes strategies related to the implementation of federal programs at the local level.
- Example: You need a key person at the local level that has the political will to implement the program (coding system: implementation mechanisms + facilitator).
- **Subcodes:**  facilitator; barrier

1. **Economic and social drivers**

- Includes information on economic and social drivers that are either facilitating of hindering access and use of health services by migrant women.
- Example: There was a restructuring on how cancer medications were bought at the federal level, and now there is a shortage of medications in the country (coding system: economic and social drivers + barrier).
- **Subcodes:** facilitator; barrier

1. **Lenses***

- Includes information on how the use of different lenses to approach the migration phenomena either facilitating of hindering access and use of health services by migrant women.
- Examples: national security lens vs. human rights lens.

1. **Other structural drivers***

- Other drivers at the structural level
- **Subcodes:** Institutional violence; predictability; wear; data; facilitator; barrier

***Other codes***

1. **Gender**

- Includes any information about differences in exposure, health needs, and access and use of health services, amongst other, between migrant women, men, and LGBTQ+ migrant populations.
- Examples: Women are more exposed than men to sexual abuse and violence during transit. Women create more community with other women, creating informal networks, when compared to men.
- **Subcodes:** facilitator; barrier

1. **Migration as determinant**

- Includes any information about the added layer of vulnerability migrant women face, due to their immigration status, when transiting through the country and accessing or using services, amongst others.
- Examples: Migrant women experience more discrimination and objectification, compared to non-migrant women, because of their immigration status.

1. **Recommendations**

- Includes information on recommendations to improve the system of care for migrant women.

1. **Missing code**

- This code will be used when no other codes are capturing what is being said. These codes will then be discussed and new codes, if necessary, will be created.
